# Differential Interaction of Platelet-Derived Extracellular Vesicles with Leukocyte Subsets in Human Whole Blood

**DOI:** 10.1038/s41598-018-25047-x

**Published:** 2018-04-26

**Authors:** René Weiss, Marion Gröger, Sabine Rauscher, Birgit Fendl, Tanja Eichhorn, Michael B. Fischer, Andreas Spittler, Viktoria Weber

**Affiliations:** 1 Christian Doppler Laboratory for Innovative Therapy Approaches in Sepsis, Department for Health Sciences and Biomedicine, Danube University Krems, Dr.-Karl-Dorrek-Strasse 30, 3500 Krems, Austria; 2 Core Facility Imaging, Medical University of Vienna, Lazarettgasse 14, 1090 Vienna, Austria; 3 Clinic for Blood Group Serology and Transfusion Medicine, Medical University of Vienna, Währinger Gürtel 18-20, 1090 Vienna, Austria; 4 Core Facility Flow Cytometry & Surgical Research Laboratories, Medical University of Vienna, Lazarettgasse 14, 1090 Vienna, Austria

## Abstract

Secretion and exchange of biomolecules via extracellular vesicles (EVs) are crucial mechanisms in intercellular communication, and the roles of EVs in infection, inflammation, or thrombosis have been increasingly recognized. EVs have emerged as central players in immune regulation and can enhance or suppress the immune response, depending on the state of donor and recipient cells. We investigated the interaction of blood cell-derived EVs with leukocyte subpopulations (monocytes and their subsets, granulocytes, B cells, T cells, and NK cells) directly in whole blood using a combination of flow cytometry, imaging flow cytometry, cell sorting, and high resolution confocal microscopy. Platelet-derived EVs constituted the majority of circulating EVs and were preferentially associated with granulocytes and monocytes, while they scarcely interacted with lymphocytes. Further flow cytometric differentiation of monocyte subsets provided clear indications for a preferential association of platelet-derived EVs with intermediate (CD14^++^CD16^+^) monocytes in whole blood.

## Introduction

Extracellular vesicles (EVs), a heterogeneous group of vesicles spanning a size of 30 nm to 3,000 nm and classified into exosomes, microvesicles, and apoptotic bodies, have been identified in all body fluids and have been recognized as important intercellular transmitters of biological signals under physiological conditions as well as in pathological settings, such as inflammation, thrombosis, neurodegenerative disorders, and cancer^1–7^. Accumulating evidence suggests a crucial and dichotomic role of EVs in immune regulation, as they may either enhance or suppress the immune response, depending on their cells of origin, their membrane properties, and their cargo^8–12^. They are capable of modulating the immune response by delivering tumor- or pathogen-derived antigens to antigen presenting cells^13,14^ or via their exposure of pro-apoptotic molecules, such as TRAIL or Galectin-9^15,16^. Furthermore, the transfer of microRNAs, small non-coding RNAs which regulate gene expression by targeting specific mRNAs and inhibiting their translation^17^ has been suggested as a major mechanism of EV-induced immune regulation^18–21^, which is either dependent on cell surface signaling^22^ or linked to the ability of immune effector cells for EV uptake^8^. The majority of studies on immunomodulating effects of EVs have been conducted in unfractionated peripheral blood mononuclear cells or in enriched cell populations, such as monocytes, monocyte-derived macrophages, T cells, B cells, and NK cells^8,23–25^, with mesenchymal stem cells (MSCs) as the most attractive source of EVs with respect to potential therapeutic applications^9,10,12^. Previous studies from our group to characterize EVs and their interactions with immune cells directly in whole blood provided evidence for a preferential association of platelet-derived EVs with granulocytes and monocytes^26^. While the cellular origin of EVs was well characterized in whole blood according to their exposure of phosphatidylserine and cell-specific surface markers, the recipient immune cells, in particular monocyte subpopulations, remained incompletely differentiated.

Human monocytes display considerable heterogeneity in the circulation regarding their phenotype and function^27–29^, and three monocyte subsets in dynamic equilibrium^30^ have been discriminated based on their surface expression of CD14 and CD16^31^. Classical monocytes (CD14^++^CD16^−^; 80–90% of all monocytes) are predominantly predetermined into the phagocytic lineage, whereas non-classical monocytes (CD14^+^CD16^++^; 2–11%) exhibit pro-inflammatory characteristics with increased antigen presentation, and intermediate monocytes (CD14^++^CD16^+^; 2–8%) have been associated with both, phagocytic and inflammatory properties^32–34^. There is increasing evidence that the distribution of these subsets along their continuum is altered in a number of diseases, including cardiovascular pathologies^35^ and sepsis^36^, but the molecular mechanisms underlying these alterations remain to be elucidated.

Characterization and enrichment of EVs are influenced by a number of pre-analytical variables^26,37–43^ and require a combination of approaches, depending on the sample matrix, the experimental purpose, as well as the further use of the EVs. This has stimulated joint efforts of scientific societies such as the ISEV, ISTH, and ISAC towards the development of well-defined procedures for EV characterization in different matrices^44^. Here, we used a combination of flow cytometry, imaging flow cytometry, and high resolution confocal microscopy for EV characterization directly in whole blood to test the hypothesis that platelet-derived EVs interact differentially with immune cells, particularly with monocyte subsets.

## Results

### Release of Platelet-Derived Extracellular Vesicles in Stored Human Whole Blood

**Figure 1:**
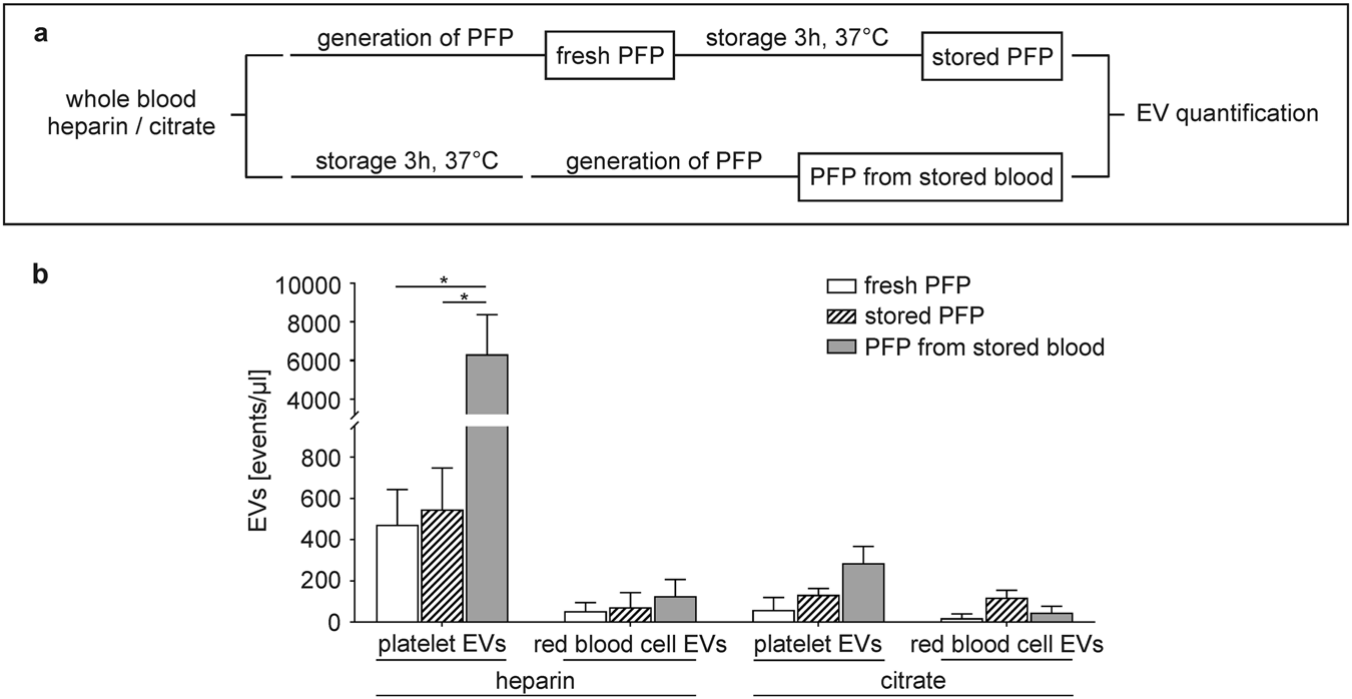
Release of extracellular vesicles (EVs) during storage of whole blood *vs*. platelet-free plasma (PFP). **(a)** Scheme summarizing the experimental set-up. Freshly drawn whole blood anticoagulated with heparin or citrate was either centrifuged immediately to obtain fresh PFP, or stored (3 h, 37 °C) prior to the generation of PFP. For comparison, PFP was stored (3 h, 37 °C) prior to analysis. EVs were characterized by flow cytometry using CD41 as marker for platelet origin, CD235a as marker for red blood cell origin, and lactadherin as marker for phosphatidylserine exposure as described in the Methods section. **(b)** Quantification of EVs in fresh PFP, stored PFP, and in PFP from stored whole blood revealed a significant release of platelet-derived EVs during the storage of heparinized whole blood (n = 3, p ≤ 0.05; paired *t*-test). Representative flow cytometry scatter plots are given in Supplementary Fig. S2.

The analysis of EVs in whole blood allows for the characterization of both, free and cell-bound EVs, but is prone to the influence of pre-analytical parameters and to the release of vesicles from blood cells, in particular from platelets, after blood sampling and storage^26,37,38^. To address these parameters, we initially assessed changes in EV counts induced during storage of whole blood or of platelet-free plasma (PFP) anticoagulated with heparin or citrate. Quantification of EVs and characterization of their cellular origin were performed by flow cytometry as previously described^45,46^ and as shown in Supplementary Fig. S1. EV counts remained stable over time in PFP obtained from freshly drawn whole blood, irrespective of the anticoagulant used, as shown in Fig. 1 (473 ± 170 *vs*. 547 ± 201 platelet EVs/µl at 0 h and 3 h, respectively, for heparin; 60 ± 60 *vs*. 133 ± 31 platelet EVs/µl at 0 h and 3 h for citrate). PFP obtained from stored heparinized whole blood, however, contained significantly elevated platelet EV counts (6,307 ± 2,068 EVs/µl; p ≤ 0.05, paired *t*-test), while red blood cell EVs remained stable. In contrast to heparinized whole blood, only a minor increase in platelet EV counts was observed upon storage of citrated whole blood (287 ± 81 EVs/µl after 3 h). Representative flow cytometry scatter plots showing the release of EVs during storage of whole blood are shown in Supplementary Fig. S2.

### Extracellular Vesicles are Mainly Associated with Monocytes and Granulocytes in Whole Blood

**Figure 2:**
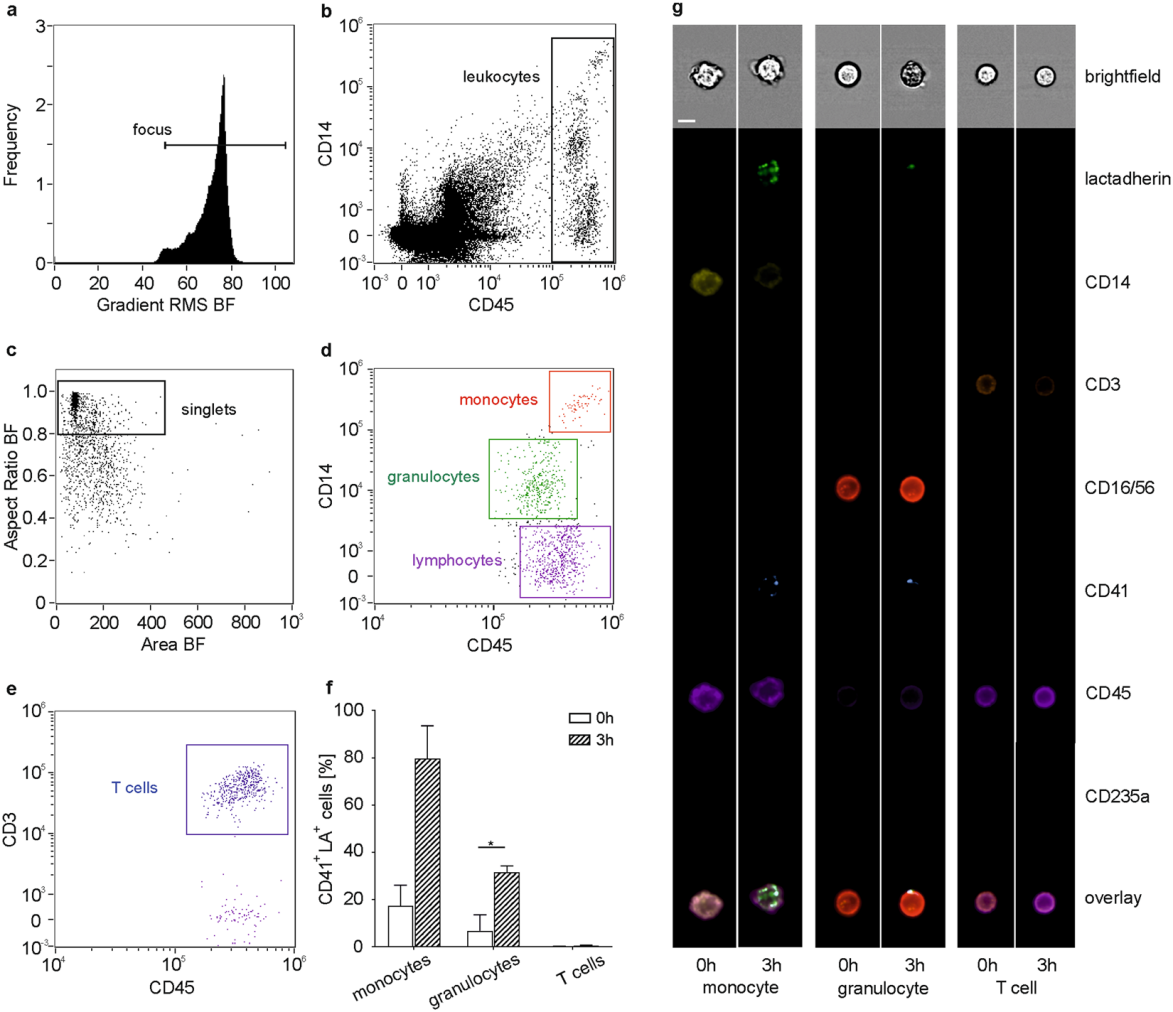
Visualization of EV-immune cell interactions using imaging flow cytometry. Imaging flow cytometry was performed after staining of blood cells using CD45-PB and CD14-PE as monocyte markers, CD16/56-PC5 as granulocyte and NK cell marker, and CD3-ECD as T cell marker. EVs were traced with lactadherin (LA)-FITC and CD41-PC7 for platelet EVs as well as with lactadherin-FITC and CD235a-APC-AF750 for red blood cell EVs as described in the Methods section. **(a)** Cells in focus were selected (gradient RMS_BF > 50); **(b)** leukocytes were identified within the focused events based on their expression patterns of CD45 and CD14; **(c)** a scatter plot of aspect ratio and area in bright field (BF) was used to define single cells within double positive focused events (aspect ratio > 0.8); **(d)** monocytes, granulocytes, and lymphocytes were identified based on their expression of CD45 and CD14 as described in the Methods section; **(e)** T cells were discriminated based on their expression of CD3 *vs*. CD45; **(f)** monocytes and granulocytes interacted with platelet EVs, whereas no association of EVs with T cells was detected; **(g)** EVs were identified as CD41^+^ lactadherin^+^ events for platelet origin or CD235a^+^ lactadherin^+^ events for red blood cell origin. Statistical significance was assessed using the Wilcoxon matched-pairs signed rank test for monocytes and T cells (not normally distributed data) or paired *t*-test for granulocytes (normally distributed data). n = 3, p ≤ 0.05. The whole panel including monocytes, granulocytes, NK cells, T cells, and B cells is shown in Supplementary Fig. S2. Scale bar, 7 µm.

**Figure 3:**
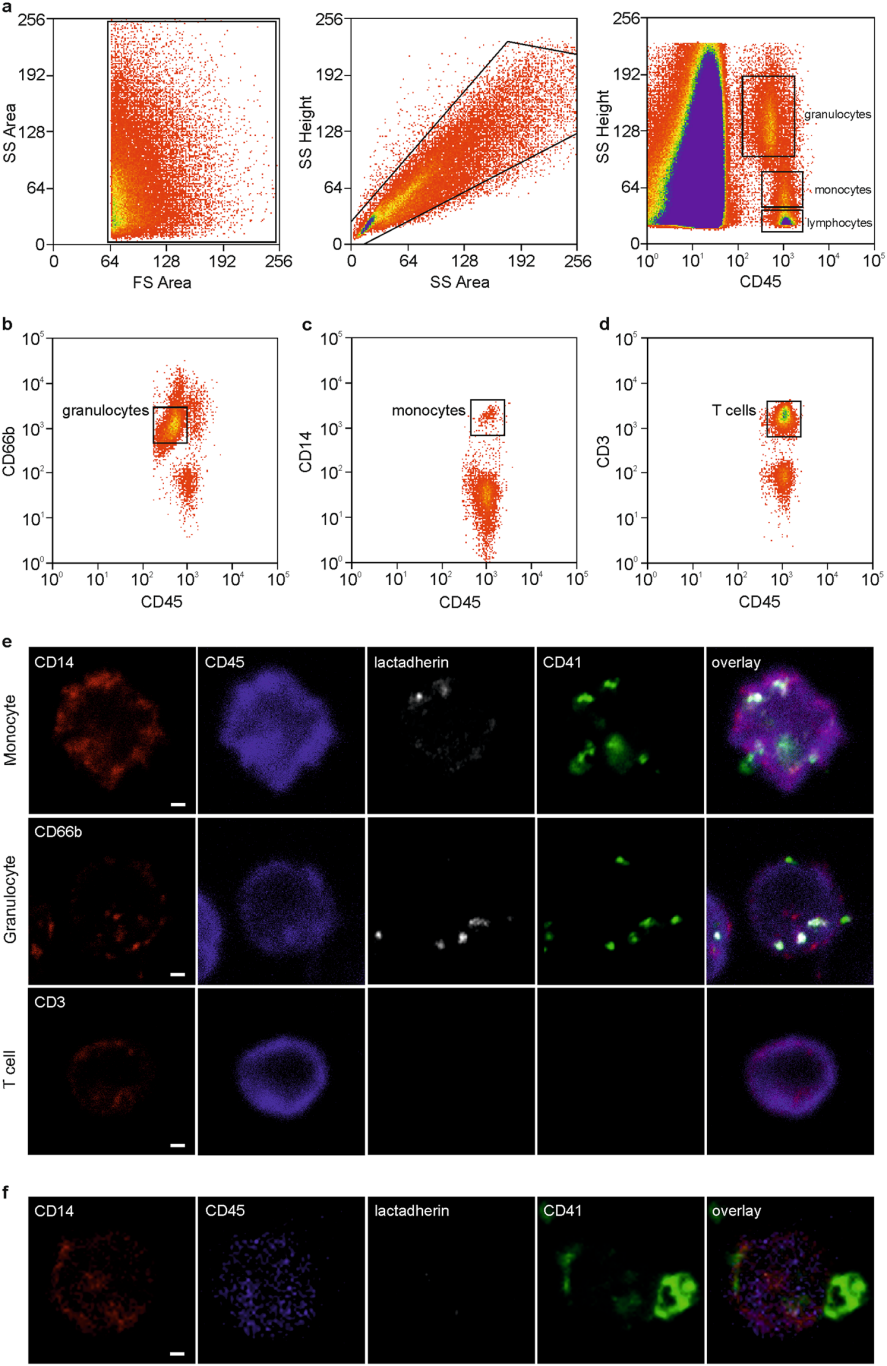
Cell sorting and high resolution confocal microscopy of EV-immune cell aggregates. To confirm the association of immune cells with EVs, monocytes, granulocytes, and T cells were sorted onto tissue culture slides, fixed, and analyzed by high resolution confocal microscopy as described in the Methods section. Prior to sorting, human whole blood (freshly drawn or stored for 3 h at 37 °C) was stained with lactadherin-AF647 and CD41-AF488 to identify platelet-derived EVs, CD45-PB as leukocyte marker, and either CD14-PE for the sorting of monocytes, CD66b-PE for the sorting of granulocytes, or CD3-PE for the sorting of T cells. **(a)** Red blood cells and platelets were excluded based on their side scatter (SS) and forward scatter (FS) area characteristics (left panel); SS height *vs*. SS area dot plots were used to exclude cell aggregates (middle panel), and granulocytes, monocytes, as well as lymphocytes were predefined in dot plots based on SS height *vs*. CD45 expression (right panel). **(b)** CD45^+^CD66b^+^ granulocytes, **(c)** CD45^+^CD14^+^ monocytes, and **(d)** CD45^+^CD3^+^ T cells were sorted onto 18-well flat bottom µ-slides, fixed, and analyzed by high resolution confocal microscopy; **(e)** representative images of sorted granulocytes, monocytes, and T cells obtained by high resolution confocal microscopy. Cells and EVs were stained as described above with CD45 (leukocyte marker; blue), CD66b (granulocyte marker; red), CD41 (platelet marker; green), and lactadherin as marker for phosphatidylserine exposing EVs (white). In the overlay, platelet EVs are visualized on the surface of and/or inside granulocytes and monocytes, but not T cells; **(f)** monocyte associated with platelets, which are clearly distinguished from platelet-derived EVs based on their size and on their exposure of phosphatidylserine. Scale bar, 1 µm.

We have previously shown the association of EVs with immune cells in human whole blood and the influence of anticoagulation and storage on this association using flow cytometry^46^ and confirmed their preferential interaction with monocytes and granulocytes using imaging flow cytometry^26^. To further specify this differential interaction of EVs with innate immune cells, we (i) developed an extended imaging flow cytometry protocol for the discrimination of monocytes, granulocytes, B cells, T cells, and NK cells (Fig. 2a–e, Supplementary Fig. S3), and (ii) used a combination of cell sorting and high resolution confocal microscopy to visualize the binding and uptake of EVs to blood cells (Fig. 3). During imaging flow cytometry, platelet EVs associated with immune cells were identified as CD41^+^lactadherin^+^ spots on the cell surface (Fig. 2g). In freshly drawn blood, 17.5 ± 8.7% of all monocytes and 6.9 ± 6.7% of all granulocytes were associated with platelet-derived EVs, while no EVs were detected on B cells, T cells, and NK cells (Fig. 2g, Supplementary Fig. S3). The percentage of monocytes and granulocytes associated with EVs increased to 79.8 ± 13.7% for monocytes, and to 31.5 ± 2.7% for granulocytes (p ≤ 0.05; paired *t*-test) upon storage of whole blood for 3 h (Fig. 2f), while B cells, T cells, and NK cells remained free from EVs. The large majority of EVs adhering to monocytes or granulocytes stained positive for CD41, indicating their platelet origin, while red blood cell-derived EVs were scarcely detectable. Isolation of monocytes, granulocytes, and T cells by cell sorting (Fig. 3a–d) onto tissue culture slides followed by high resolution confocal microscopy confirmed the preferential association of EVs with monocytes and granulocytes and revealed EV uptake by monocytes and granulocytes, while we found no evidence for the association of EVs with T cells (Fig. 3e). Based on their larger diameter and their lack of phosphatidylserine exposure, platelets were clearly distinguished from platelet EVs in high resolution confocal microscopy (Fig. 3f).

### The Distribution of Human Monocyte Subsets is Influenced by Monocyte Isolation and Storage

**Figure 4:**
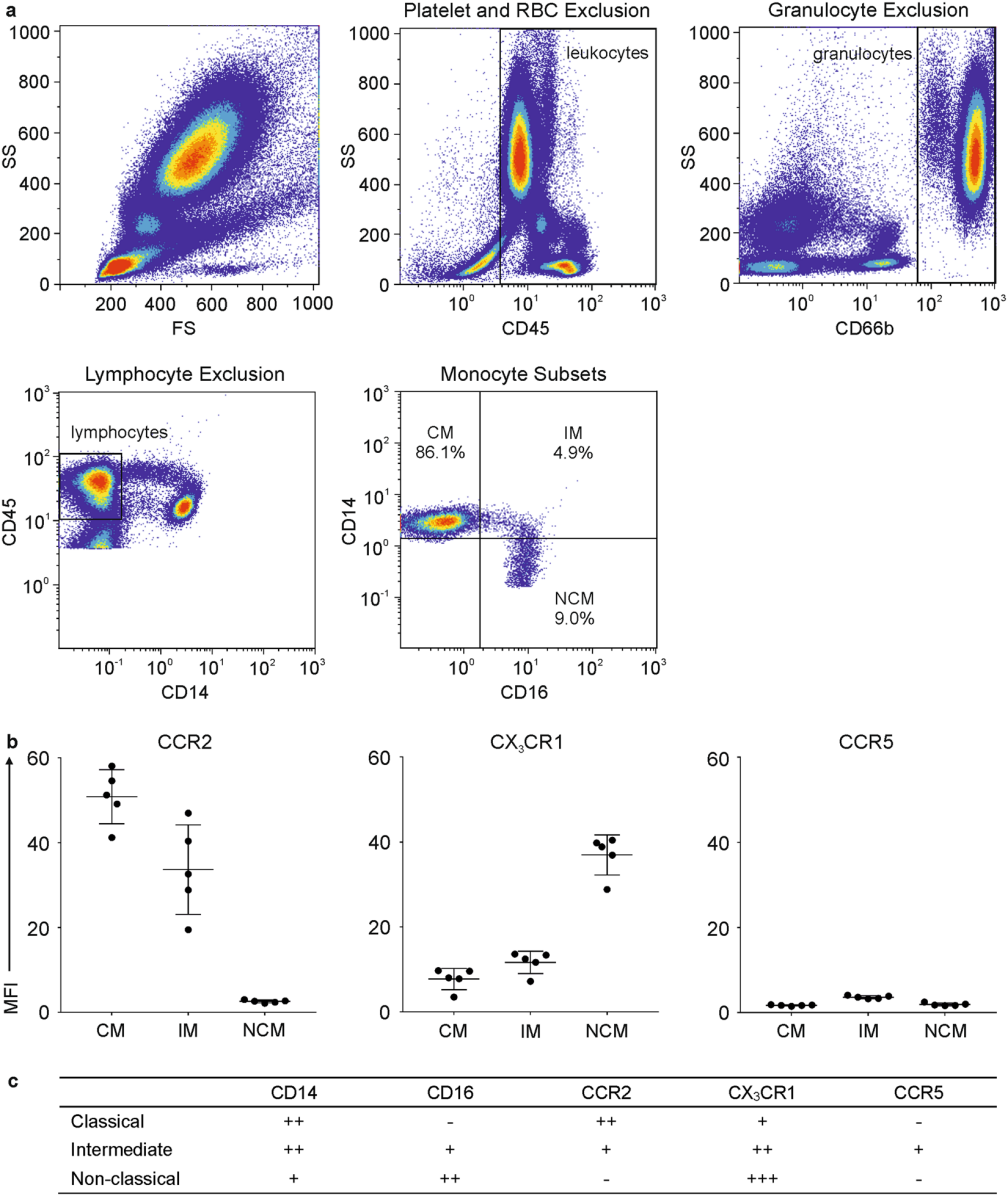
Identification and characterization of monocyte subsets in whole blood. Using flow cytometry, monocyte subsets were identified directly in whole blood based on their expression patterns of CD14 and CD16 as described in the Methods section. **(a)** Gating for the identification of monocyte subsets was based on the successive exclusion of platelets, red blood cells (RBC), granulocytes, and lymphocytes on bivariate scatter plots. The remaining population was discriminated on a CD14 *vs*. CD16 scatter plot to discriminate three monocyte subsets, classical (CM), intermediate (IM), and non-classical (NCM) monocytes (n = 5). **(b)** Monocyte subsets were further characterized based on their expression of chemokine receptors CCR2, CX_3_CR1, and CCR5 (n = 3). MFI, mean fluorescence intensity. **(c)** Phenotypic characterization of monocyte subsets based on their expression of CD14, CD16, CCR2, CX_3_CR1, and CCR5.

After demonstrating that monocytes are a preferred target of EVs in whole blood, we proceeded to investigate the interaction of EVs with monocyte subsets. Classical, intermediate, and non-classical monocytes were identified by flow cytometry directly in whole blood^47^ and discriminated based on their differential expression of CD14 and CD16 (Fig. 4a and c), as well as by their expression patterns of the chemokine receptors CCR2, CX_3_CR1, and CCR5 (Fig. 4b and c)^48–50^. Complementing their analysis in whole blood, we characterized monocyte subsets after isolation of monocytes from peripheral blood mononuclear cells (PBMCs) by negative depletion with antibody-coated magnetic beads. Freshly drawn whole blood contained 86.1 ± 2.1% classical monocytes, 4.9 ± 1.1% intermediate monocytes, and 9.0 ± 2.6% non-classical monocytes (Fig. 4a; Supplementary Fig. S4a). The distribution of monocyte subsets in freshly isolated monocyte populations did not differ from their distribution in whole blood (86.8 ± 4.0% classical monocytes, 3.6 ± 1.0% intermediate monocytes, and 9.6 ± 3.3% non-classical monocytes; Supplementary Fig. S4b). After an overnight resting phase in cell culture medium, however, the distribution of monocyte subsets shifted to 39.5 ± 24.6% classical monocytes, 59.1 ± 24.0% intermediate monocytes, and 1.4 ± 0.9% non-classical monocytes (Supplementary Fig. S4c). This shift was statistically significant for all three monocyte subsets (p ≤ 0.05; paired *t*-test).

### Classical and Intermediate Monocytes are Preferentially Associated with Extracellular Vesicles

**Figure 5:**
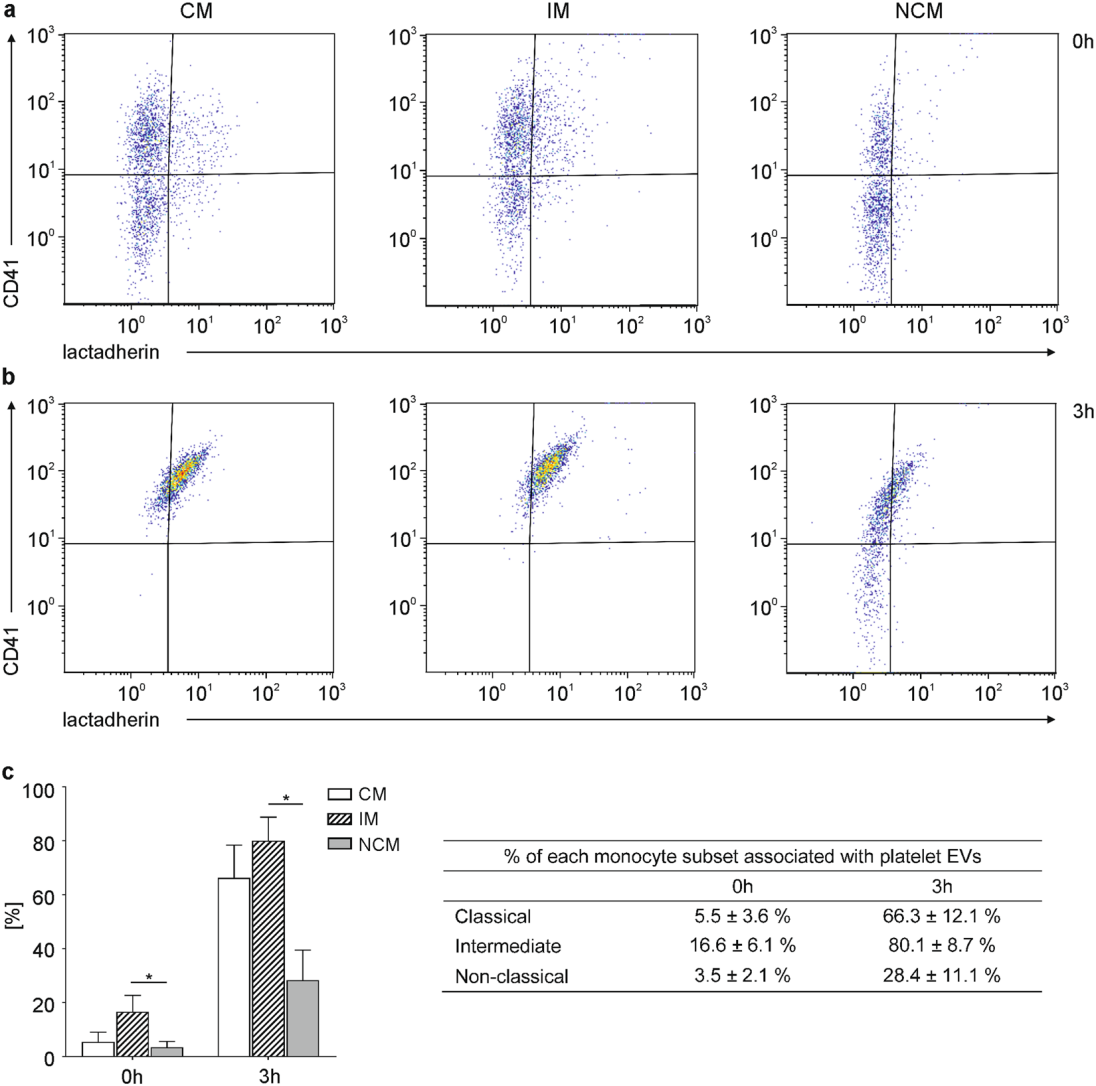
Differential interaction of EVs with monocyte subsets. Monocyte subsets were characterized using flow cytometry as shown in Fig. 4. Classical (CM), intermediate (IM), and non-classical (NCM) monocytes associated with platelet-derived EVs were identified as CD41^+^lactadherin^+^ cells **(a)** in freshly drawn whole blood and **(b)** after storage of whole blood for 3 h, as shown on representative scatter plots; **(c)** percentages of classical, intermediate, and non-classical monocytes associated with EVs (average of five independent experiments). A total of 2,000 events were recorded for each monocyte subset. At both time points, intermediate monocytes showed significantly higher association with EVs as compared to non-classical monocytes (n = 5; p ≤ 0.05; Friedman test).

Next, we investigated the association of monocyte subsets with EVs using flow cytometry. To rule out any possibility of the results being distorted by differences in the relative abundance of monocyte subsets, a total of 2,000 events were recorded for each monocyte subset. In freshly drawn blood, 5.5 ± 3.6% of all classical monocytes, 16.6 ± 6.1% of all intermediate monocytes, and 3.5 ± 2.1% of all non-classical monocytes were CD41^+^lactadherin^+^, indicating their association with platelet-derived EVs (Fig. 5a). After a 3 h storage, monocyte-EV aggregates increased to 66.3 ± 12.1% for classical monocytes, to 80.1 ± 8.7% for intermediate monocytes, and to 28.4 ± 11.1% for non-classical monocytes (Fig. 5b), providing evidence for a preferential association of EVs with intermediate monocytes in whole blood, as summarized in Fig. 5c.

## Discussion

Circulating EVs have important roles in immune regulation^51–55^ and are involved in the pathology of inflammatory and autoimmune disorders. While EVs from both, non-immune and immune cells have been shown to exert effects on isolated leukocyte populations, little is known on the interaction of EVs released by different cell types with immune cells in the circulation. Accordingly, in the current study, we characterized the association of platelet-derived EVs with immune cells directly in whole blood. This approach was mandated by the experimental purpose of our study, as EVs associated with blood cells would be depleted during plasma generation. While analysis in whole blood circumvents the activation of blood cells during centrifugation^56^, parameters related to the collection and storage of peripheral blood can induce post-sampling EV release alike, resulting in elevated EV counts. Therefore, to define the experimental conditions for further EV characterization in whole blood, we assessed the release of EVs over time in blood samples anticoagulated with heparin or citrate, using platelet-free plasma as a reference. Our data firstly demonstrate the critical impact of anticoagulation, showing that chelation of Ca^++^ by citrate limits the release of EVs^26,37,57,58^. Secondly, they indicate that platelets constitute the main source of EVs in stored whole blood, as evidenced by the exposure of CD41 on the large majority of released EVs^46,59,60^. Based on these findings, we chose heparinized whole blood as source for all further experiments to follow the association of released EVs with immune cells over time.

Our experimental approach to study this association relied on a combination of flow cytometry, imaging flow cytometry, cell sorting, and high resolution confocal microscopy. Beyond the quantification of EVs in a size range of 200 nm to 1,000 nm^7^, flow cytometry yields information on the cellular origin of EVs based on the expression of markers derived from their parent cells. While positive staining of immune cells for lactadherin or annexin V^46,60^ during flow cytometry can provide indirect evidence for their association with phosphatidylserine exposing EVs^26^, imaging flow cytometry allows for the direct visualization of labeled EVs associated with immune cells^39^. Our imaging flow cytometry data suggest that the large majority of EVs released in whole blood were platelet-derived and associated with granulocytes and monocytes. As a limitation, however, the resolution achieved with imaging flow cytometry was not entirely satisfying with respect to the discrimination of platelet-derived EVs and platelets, since they can overlap in size, and since platelets can expose phosphatidylserine upon activation. A combination of cell sorting and high resolution confocal microscopy, however, provided a clear discrimination of platelet-derived EVs (less than 1 µm in diameter, CD41^+^lactadherin^+^), and platelets (3 µm in diameter, CD41^+^lactadherin^−^), and revealed the presence of platelet-derived EVs on the surface of and inside granulocytes and monocytes. These findings are in line with published data showing that MSC-derived EVs were mostly internalized by monocytes and scarcely by lymphocytes^8–12^ upon incubation with unfractionated PBMCs.

To further address the interaction of EVs with monocytes, and given the important role of monocytes in the pathogenesis of systemic inflammatory disorders, we investigated the association of platelet-derived EVs with monocyte subsets. We relied on the direct characterization of monocyte subsets in whole blood using flow cytometry, employing a gating strategy based on the sequential exclusion of platelets, remaining red blood cells, granulocytes, and lymphocytes^47^. Classical, intermediate, and non-classical monocytes were identified based on their differential expression of CD14 and CD16, as well as by their differential CCR2, CX_3_CR1, and CCR5 chemokine receptor expression^34^. Complementing their characterization in whole blood, we analyzed the distribution of monocyte subsets using isolated monocytes (i) immediately after their isolation from whole blood and (ii) after an overnight resting phase of the isolated monocytes in cell culture medium supplemented with 10% AB serum. Analysis of monocyte subsets in whole blood and immediately after monocyte isolation yielded almost identical subset distributions. Notably, while the antibody panel used to label and deplete non-monocytes during monocyte isolation contained anti-CD16 antibodies to tag granulocytes, this did not result in depletion of CD16^+^ monocytes, suggesting that granulocytes were preferentially labelled due to their strong CD16 expression. Overnight resting of the isolated monocytes on cell culture plates, however, induced a pronounced shift towards intermediate monocytes, which increased from less than 5% to almost 60% of all monocytes. One could speculate that traces of lipopolysaccharide (LPS) contained in the gradient centrifugation medium might have induced these alterations in monocyte subset distribution, since LPS contaminations in media or recombinant proteins have been identified as cause of monocyte activation in a number of studies^61^. Still, a limulus amoebocyte lysate assay remained negative and we therefore do not have evidence for the presence of even trace amounts of LPS in our experimental setting.

Regarding the association of EVs with monocyte subsets, intermediate monocytes were the main subset associated with EVs in freshly drawn blood. Storage of whole blood induced the release of platelet-derived EVs and enhanced their association with all subsets, with the highest proportion of EV-monocyte aggregates for intermediate monocytes after 3 h. As to the mechanism of interaction between EVs and monocyte subsets, one could speculate that activated CD11b, a member of the β-integrin family, which is strongly upregulated on intermediate monocytes in low-grade inflammation^62^, may be involved in this differential interaction. Indeed, a recent study demonstrated that phagocytosis of red blood cell-derived EVs was partially abrogated after co-incubation with antibodies against CD11b and CD18^63^. Alternatively, the tyrosine kinase receptors Tyro3, Axl, and Mer (TAMs) and their ligands protein S and Gas6 might have a role in mediating the association of EVs with immune cells, as elegantly shown for endothelial uptake of platelet-derived EVs, which is mediated by Axl and its ligand Gas6^64^.

In conclusion, we demonstrate (i) that platelet EVs interact differentially with leukocyte subsets and are preferentially associated with monocytes and granulocytes in human whole blood, while (ii) they are rarely detected in association with lymphocytes, and we provide evidence that (iii) platelet EVs are predominantly associated with classical and intermediate monocyte subsets. Moreover, our data support the notion that the distribution of classical, intermediate, and non-classical monocytes is influenced by monocyte isolation and storage, with a clear shift towards CD14^++^CD16^+^ intermediate monocytes during storage of isolated monocyte populations.

## Methods

### Human Blood and Plasma

Venous blood was collected from healthy adult volunteers into tubes (Vacuette, Greiner Bio-One, Kremsmuenster, Austria) containing sodium heparin or citrate as stated for the individual experiments using a 21 gauge needle. Blood collection was approved by the Review Board of Danube University Krems, and written informed consent was obtained from all donors. The first 3 ml of blood were discarded or used to determine the blood count (KX-21 N, Sysmex, Neumuenster, Germany). Platelet-poor plasma was obtained from whole blood by centrifugation at 2,500 g for 10 min. Platelet-free plasma (PFP) was generated by centrifugation of platelet-poor plasma at 13,000 g for 15 min. All centrifugation steps were carried out at room temperature (RT).

### Cell Culture Media and Reagents

Phosphate buffered saline (PBS) without calcium and magnesium was obtained from Life Technologies (Paisley, UK) and filtered through 0.1 µm filters (Merck Millipore, Billerica, MA) immediately before use. RPMI-1640 (RPMI) and ethylene diamine tetraacetic acid disodium salt (EDTA) were purchased from Sigma Aldrich (St Louis, MO). All monoclonal antibody-fluorochrome conjugates used for flow cytometry, imaging flow cytometry, cell sorting, and confocal microscopy are listed in Supplementary Table 1.

### Flow Cytometric Characterization of Extracellular Vesicles

EVs were characterized in PFP, which was generated from freshly drawn whole blood as described above. Samples were diluted 1:400 in sterile-filtered PBS prior to analysis. Staining of EVs was performed for 15 min in the dark using FITC-conjugated lactadherin as marker for phosphatidylserine in combination with CD41-PC7 and CD235a-APC-AF750 as markers of platelet and red blood cell origin, respectively. All antibodies were centrifuged at 17,000 g for 10 min at 4 °C prior to use to remove precipitates. Flow cytometry was performed using a CytoFLEX flow cytometer (Beckman Coulter, Brea, CA) equipped with 405 nm, 488 nm, 561 nm, and 638 nm lasers. The triggering signal was set to the side scatter (SS) of the 405 nm laser and was combined with triggering on fluorescence. Fluorescent-green silica particles (1 µm; excitation/emission 485/510 nm; Kisker Biotech, Steinfurt, Germany) were used for calibration, and the EV gate was set below the 1 µm bead cloud as indicated in Supplementary Fig. S1a and b. Samples were acquired for 2 min at a flow rate of 10 µl/min. To avoid carry-over effects, a 2 min washing step with sterile filtered double distilled water was performed between each measurement at a flow rate of 60 µl/min. Data were analyzed using the CytExpert Software Version 1.2 (Beckman Coulter). Buffer controls and fluorochrome labeled reagent controls are shown in Supplementary Fig. S1c–f. The release of EVs during storage of whole blood and PFP was compared by analyzing EVs in freshly drawn PFP and after storage of PFP for 3 h at 37 °C with gentle agitation. Alternatively, PFP was obtained after storage of whole blood (3 h, 37 °C), and analyzed as described above, as shown in Fig. 1 and in Supplementary Fig. S2 (n = 3).

### Imaging Flow Cytometry

In addition to their flow cytometric characterization in plasma, EVs were analyzed directly in heparinized whole blood and their interaction with blood cells was assessed by Imaging Flow Cytometry (ImageStream^x^MkII cytometer, INSPIRE v6.1, Amnis, EMD Millipore, Seattle, WA)^26^. Analysis was performed at low flow rate and high sensitivity using 60-fold magnification, providing a pixel size of 0.3 µm × 0.3 µm. Whole blood was analyzed at a final dilution of 1:100 in PBS after staining of EVs and blood cells for 15 min with lactadherin-FITC as marker of phosphatidylserine exposing EVs, CD45-PB and CD14-PE as monocyte markers, CD16/CD56-PC5 as granulocyte and NK cell markers, CD3-ECD as marker for T cells, CD41-PC7 as platelet marker, and CD235a-APC-AF750 as red blood cell marker. Events were first selected on focused events (Gradient RMS_BF > 50). Subsequently, singlets were gated *vs*. doublets in a new plot using Area_BF (Ch01) *vs*. Aspect Ratio_BF (Ch01). Singlets were defined visually and correlated with an aspect ratio > 0.8. Singlets were further plotted to CD14-PE (Intensity-MC-Ch03) *vs*. CD45-PB (Intensity-MC-Ch07) to identify monocytes (CD45^+^CD14^++^), granulocytes (CD45^+^CD14^+^), and lymphocytes (CD45^+^CD14^−^) as well as T cells (CD45^+^CD14^–^CD3^+^). Within the monocyte gate, lactadherin-FITC (Intensity-MC-Ch02) was plotted against CD41-PC7 (Intensity-MC-Ch06) to identify monocyte carrying lactadherin^+^ EVs of platelet origin, and lactadherin-FITC was plotted against CD235a-APC-AF750 (Intensity-MC-Ch12) for identification of monocytes carrying red blood cell-derived EVs. An analogous gating strategy was used to identify interactions of EVs with granulocytes and T cells. For calculation of the spectral overlap, single stained controls were run for each fluorochrome. EVs appeared as small, spherical lactadherin^+^ events associated with monocytes and granulocytes (n = 3).

### Cell Sorting

To confirm the association of blood cells with EVs, monocytes, granulocytes, as well as T cells were sorted onto tissue culture slides as described below, fixed, and analyzed by high resolution confocal microscopy. Human whole blood was stained for 15 min with lactadherin-AF647, CD45-PB, and CD41-AF488, and either CD14-PE for the sorting of monocytes, CD66b-PE for the sorting of granulocytes, and CD3-PE for the sorting of T cells. Stained blood was diluted 1:10 with PBS, and cell sorting was performed using a MoFloAstrios EQ (Beckman Coulter). To exclude red blood cells and platelets, cells were depicted on a dot plot using side scatter (SS) against CD45. CD45^+^ cells were gated and divided into a CD45^+^/CD14^+^ plot, a CD45^+^/CD66b^+^ plot and a CD45^+^/CD3^+^ plot. After color compensation, CD45^+^CD14^+^ monocytes, CD45^+^CD66b^+^ granulocytes, and CD45^+^CD3^+^ T cells were sorted onto 18-well flat bottom µ-slides treated for tissue culture (ibidi, Martinsried, Germany). Just before sorting, side streams were adjusted to fit exactly into the wells. Wells were prefilled with fixation solution containing 7.5% formaldehyde (Liquid Productions GmbH, Flintsbach, Germany) to prepare cells for high resolution imaging. In total, 5,000 cells of each population were sorted in purity mode (n = 5).

### High Resolution Confocal Microscopy

Labeled cells were sorted onto tissue culture slides and fixed as described above. Previous to imaging, cells were allowed to rest at 4 °C for 15 min, followed by washing with PBS and addition of mounting medium (ibidi). Image acquisition was performed on an LSM780 Laser Scanning Microscope (Carl Zeiss, Oberkochen, Germany) at excitation wavelengths of 405 nm, 488 nm, 560 nm, and 633 nm. Confocal superresolution images were acquired using the Airy Scan Module from Zeiss^65^. Image analysis was performed using the ZEN 2.3 (blue edition) software (Carl Zeiss).

### Flow Cytometric Characterization of Monocyte Subsets in Whole Blood

For flow cytometric characterization of classical, intermediate, and non-classical monocytes directly in whole blood^47^, freshly drawn heparinized blood (200 µl) was stained with CD14-PE, CD16-PC5, CD66b-APC, and CD45-PB for 15 min and treated with 2 ml of lysis solution (IOTest 3, Beckman Coulter) for 10 min at RT to lyse red blood cells. The remaining cells were pelleted for 5 min at 150 g, washed with PBS, resuspended in 500 µl PBS, and analyzed on a Gallios flow cytometer (Beckman Coulter) equipped with 405 nm, 488 nm, and 638 nm lasers. Data were analyzed using the Kaluza Software (Beckman Coulter). For gating, platelets and remaining red blood cells were excluded based on their CD45 expression, granulocytes were excluded based on their CD66b expression, and lymphocytes were excluded based on their expression of CD45 and CD14. Monocyte subsets were identified based on their expression of CD14 and CD16 as classical monocytes (CD14^++^CD16^−^), intermediate monocytes (CD14^++^CD16^+^), and non-classical monocytes (CD14^+^CD16^++^)^47^ (n = 5). In addition to CD14 and CD16 surface expression, monocyte subsets were characterized via the exposure of chemokine receptors CCR2, CX_3_CR1, CCR5 as summarized in Fig. 4c^48,49^ (n = 3).

### Flow Cytometric Characterization of Monocytes after Isolation

Freshly drawn whole blood was diluted 1:2 in PBS containing 5 mM EDTA, and peripheral blood mononuclear cells (PBMCs) were isolated by density gradient centrifugation on Ficoll-Paque PLUS (GE Healthcare, Uppsala, Sweden) as previously described^66^. Monocytes were isolated from PBMCs by negative depletion of non-monocytes via indirect magnetic labeling with antibodies against CD3, CD7, CD16, CD19, CD56, CD123, and CD235a (Monocyte Isolation Kit II, Miltenyi Biotec, Bergisch Gladbach, Germany). Isolated monocytes were resuspended in PBS (5 × 10^5^ cells/100 µl), stained, and analyzed using flow cytometry to discriminate monocyte subsets as described above. Analysis was performed either directly after monocyte isolation or following an overnight resting phase in 12-well suspension culture plates (Greiner) at 37 °C, 5% CO_2_ in RPMI medium containing 10% sterile filtered AB serum. For flow cytometry, cells were detached from the plates by incubation with PBS containing 1.5 mM EDTA for 5 min (n = 5).

### Interaction of Monocyte Subsets with Extracellular Vesicles

The interaction of EVs with monocyte subsets was analyzed directly in whole blood using the Gallios flow cytometer (Beckman Coulter). Monocyte subsets were identified based on their expression of CD14 and CD16 as described above. For each monocyte subset, 2,000 events were acquired, and platelet-derived EVs associated with monocyte subsets were traced using lactadherin-FITC and CD41-PC7 (n = 5).

### Statistical Analysis

Statistical analysis was performed using GraphPad Prism version 7.02 (La Jolla, CA). Data are presented as means ± standard deviation. For normally distributed data, the paired *t*-test was applied to analyze differences between time points, while the non-parametric Wilcoxon matched-pairs signed rank test was used for analysis of not normally distributed data. Friedman test followed by Dunn’s multiple comparisons test was performed to analyze differences in monocyte subsets. Significance was accepted at p ≤ 0.05.

## Electronic supplementary material

Supplementary Information
