# Supplementary material for: Differential Interaction of Platelet-Derived Extracellular Vesicles with Leukocyte Subsets in Human Whole Blood
Source: Sci Rep. 2018 Apr 26;8:6598. doi: 10.1038/s41598-018-25047-x (PMC5920058; doi:10.1038/s41598-018-25047-x)
Supplement: Supplementary file 1 — Supplementary Information [file 41598_2018_25047_MOESM1_ESM.pdf]

Differential Interaction of Platelet-Derived Extracellular Vesicles with Leukocyte Subsets in Human Whole Blood

René Weiss[1], Marion Gröger[2], Sabine Rauscher[2], Birgit Fendl[1], Tanja Eichhorn[1], Michael B. Fischer[1,3], Andreas Spittler[4#], Viktoria Weber[1#]

[1]Christian Doppler Laboratory for Innovative Therapy Approaches in Sepsis, Department for Health Sciences and Biomedicine, Danube University Krems, Dr.-Karl-Dorrek-Strasse 30, 3500 Krems, Austria

[2]Core Facility Imaging, Medical University of Vienna, Lazarettgasse 14, 1090 Vienna, Austria

[3]Clinic for Blood Group Serology and Transfusion Medicine, Medical University of Vienna, Währinger Gürtel 18-20, 1090, Vienna, Austria

[4]Core Facility Flow Cytometry & Surgical Research Laboratories, Medical University of Vienna, Lazarettgasse 14, 1090 Vienna, Austria

[#]equally contributing last authors

To whom correspondence should be addressed: Prof. Viktoria Weber, Danube University Krems, Dr.-Karl-Dorrek-Strasse 30, 3500 Krems, Austria, Tel.: +43 2732 893 2632; Fax: +43 2732 893 4600; email: viktoria.weber@donau-uni.ac.at

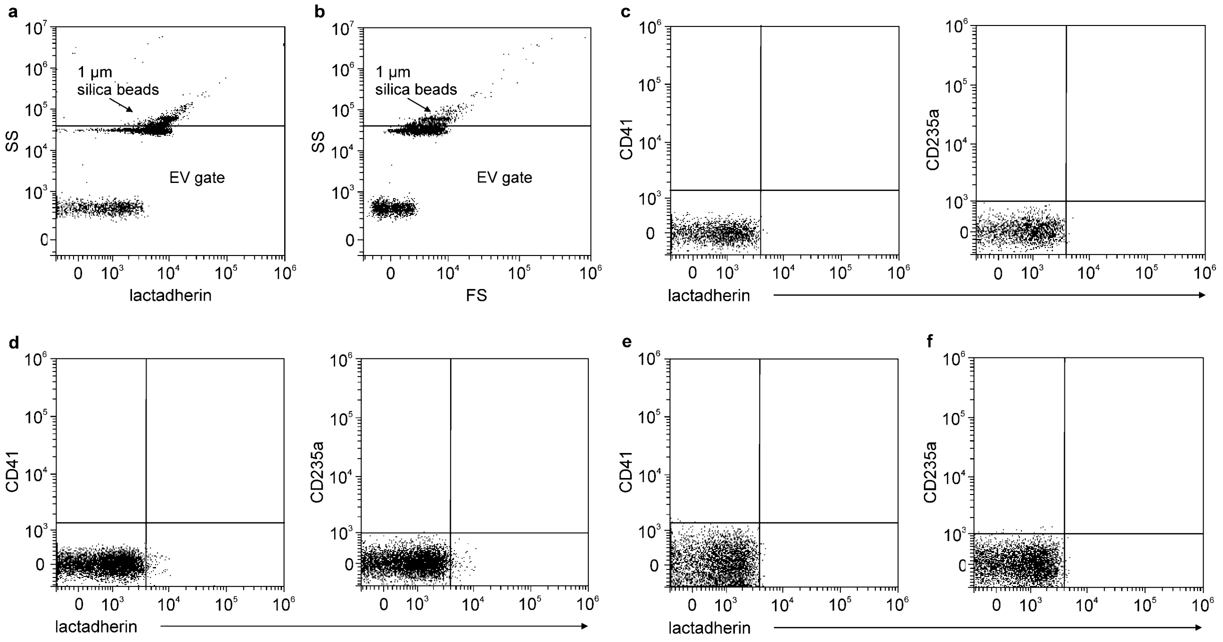

Supplementary Figure S1. Flow cytometric characterization of extracellular vesicles (EVs). (a) Flow cytometric characterization of extracellular vesicles was performed after calibration with 1 µm fluorescent-green silica particles (excitation/emission 485/510 nm), and the EV gate was set below the 1 µm bead cloud as indicated **(a)** in the side scatter (SS) *vs.* lactadherin dot plot and **(b)** in the SS *vs.* forward scatter (FS) dot plot. The following buffer controls were included: **(c)** unstained phosphate-buffered saline (PBS); **(d)** PBS stained with FITC-labeled lactadherin; **(e)** PBS stained with PC7-labeled CD41; **(f)** PBS stained with APC-AF750-labeled CD235a (n=3). All antibody-fluorochrome conjugates are specified in Supplementary Table 1.

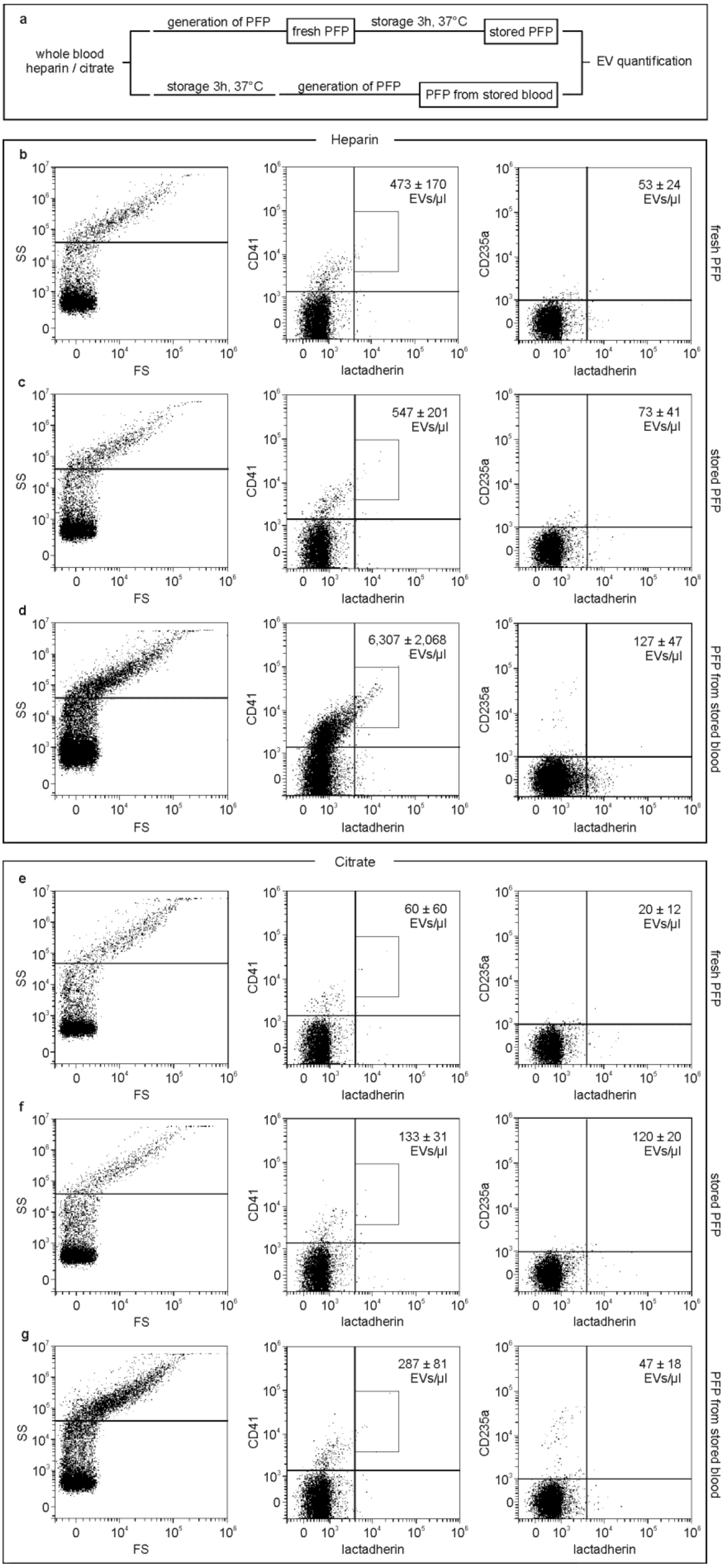
a
whole blood
heparin / citrate
generation of PFP
fresh PFP
storage 3h, 37°C
stored PFP
storage 3h, 37°C
generation of PFP
PFP from stored blood
EV quantification
Heparin
b
fresh PFP
473 ± 170 EVs/µl
53 ± 24 EVs/µl
c
stored PFP
547 ± 201 EVs/µl
73 ± 41 EVs/µl
d
PFP from stored blood
6,307 ± 2,068 EVs/µl
127 ± 47 EVs/µl
Citrate
e
fresh PFP
60 ± 60 EVs/µl
20 ± 12 EVs/µl
f
stored PFP
133 ± 31 EVs/µl
120 ± 20 EVs/µl
g
PFP from stored blood
287 ± 81 EVs/µl
47 ± 18 EVs/µl
SS
FS
CD41
CD235a
lactadherin

Supplementary Figure S2. Release of extracellular vesicles during storage of whole blood *vs.* platelet free plasma (PFP). (a) Scheme of the experimental set-up. Freshly drawn whole blood anticoagulated with heparin or citrate was either centrifuged immediately to obtain fresh PFP, or stored (3h, 37°C) prior to the generation of PFP. For comparison, PFP was stored (3h, 37°C) prior to analysis. EVs were characterized by flow cytometry using CD41 as marker for platelet origin, CD235a as marker for red blood cell origin, and lactadherin as marker for phosphatidylserine exposure as described in the Methods section. **(b-g)** Representative side scatter (SS) *vs.* forward scatter (FS) dot plots as well as CD41 *vs.* lactadherin and CD235a *vs.* lactadherin dot plots are shown for fresh PFP (**b, e**) or stored PFP (**c, f**). For comparison, PFP from stored whole blood was analyzed (**d, g**), revealing a pronounced release of platelet-derived EVs. A bar chart summarizing the data of three independent experiments is shown in Fig. 1b in the main manuscript (n=3).

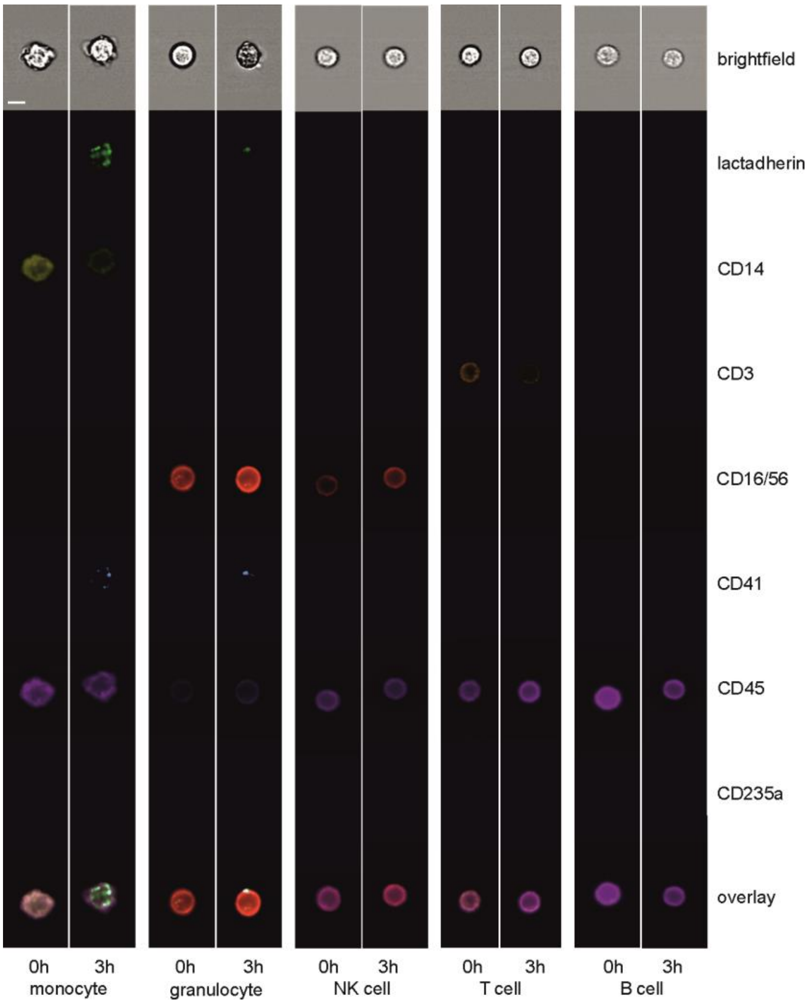

Supplementary Figure S3. Visualization of extracellular vesicle-immune cell aggregates using imaging flow cytometry. The association of EVs with immune cells was analyzed by imaging flow cytometry as described in the Methods section after staining with CD45-PB and CD14-PE as monocyte markers, CD16/56-PC5 as granulocyte and NK cell marker, and CD3-ECD as T cell marker. Monocytes and granulocytes interacted with platelet EVs, whereas no association of EVs and NK cells, T cells, or B cells was detected. EVs appeared as spots on the cell surface and were identified as $CD41^{+}lactadherin^{+}$ events for platelet origin or $CD235a^{+}lactadherin^{+}$ events for red blood cell origin. Scale bar, 7 µm. All antibody-fluorochrome conjugates are specified in Supplementary Table 1.

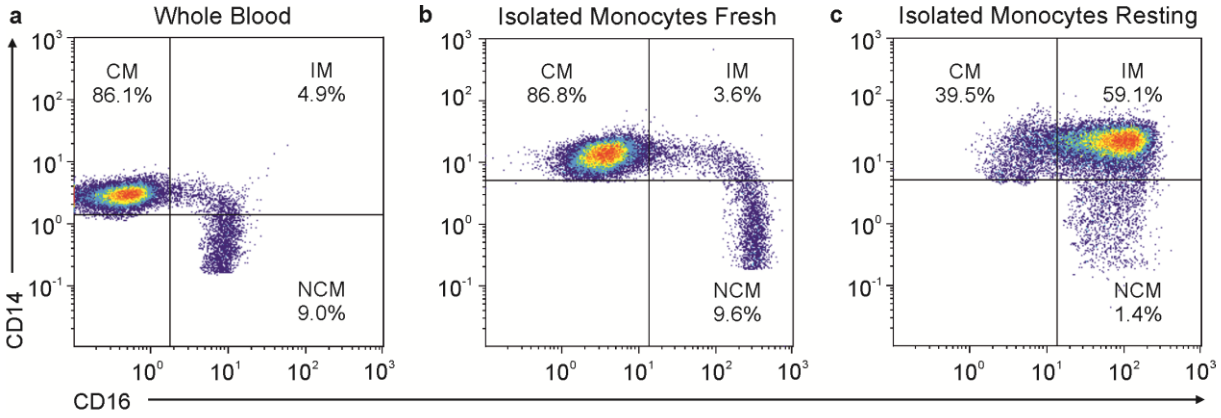

Supplementary Figure S4: Characterization of monocyte subsets in whole blood and after monocyte isolation. Monocyte subsets were identified based on their expression patterns of CD14 and CD16 either directly in whole blood or after isolation by gradient centrifugation and negative depletion as described in the Methods section to identify classical (CM), intermediate (IM), and non-classical (NCM) monocytes. **(a)** characterization of monocyte subsets in whole blood; **(b)** monocyte subset distribution directly after monocyte isolation; **(c)** monocyte subset distribution after an overnight resting phase. The latter resulted in a statistically significant decrease in classical monocytes and a statistically significant increase in intermediate monocytes, as shown using a paired *t*-test (n=5, $p \leq 0.05$).

Supplementary Table 1. Antibodies used for the staining of cell surface antigens.

Flow Cytometry and Imaging Flow Cytometry					
Antigen	Clone	Marker for	Fluorochrome	Abbreviation	Supplier
CD3	UCHT1	T cells	Phycoerythrin Texas Red-X	ECD	Beckman Coulter
CD14	RMO52	monocytes	Phycoerythrin	PE	Beckman Coulter
CD16	3G8	granulocytes	Phycoerythrin Cyanin 5.1	PC5	Beckman Coulter
CD56	N901	NK cells	Phycoerythrin Cyanin 5.1	PC5	Beckman Coulter
CD41	P2	platelets	Phycoerythrin Cyanin 7	PC7	Beckman Coulter
CD45	J33	leukocytes	Pacific Blue	PB	Beckman Coulter
CD235a	HIR2 (GA-R2)	erythrocytes	Allophycocyanin Alexa Fluor 750	APC-AF750	Beckman Coulter
lactadherin	-	phosphatidylserine	Fluorescein Isothiocyanate	FITC	Haematologic Technologies, Inc.

Sorting and Confocal Microscopy					
Antigen	Clone	Marker for	Fluorochrome	Abbreviation	Supplier
CD3	OKT3	T cells	Phycoerythrin	PE	Beckman Coulter
CD14	RMO52	monocytes	Phycoerythrin	PE	Beckman Coulter
CD41	MEM06	platelets	Alexa Fluor 488	AF488	Abcam
CD45	J33	leukocytes	Pacific Blue	PB	Beckman Coulter
CD66b	REA306	granulocytes	Phycoerythrin	PE	Miltenyi
lactadherin	-	phosphatidylserine	Alexa Fluor 647	AF647	Haematologic Technologies, Inc.

Characterization of Monocyte Subsets					
Antigen	Clone	Marker for	Fluorochrome	Abbreviation	Supplier
CD14	RMO52	monocytes	Phycoerythrin	PE	Beckman Coulter
CD16	3G8	monocytes	Phycoerythrin Cyanin 5.1	PC5	Beckman Coulter
CD41	P2	platelets	Phycoerythrin Cyanin 7	PC7	Beckman Coulter
CD45	J33	leukocytes	Pacific Blue	PB	Beckman Coulter
CD66b	80H3	granulocytes	Allophycocyanin	APC	Beckman Coulter
lactadherin	-	phosphatidylserine	Fluorescein Isothiocyanate	FITC	Haematologic Technologies, Inc.
CCR2	K036C2	monocytes	Fluorescein Isothiocyanate	FITC	Biolegend
CX_3CR1	2A9-1	monocytes	Fluorescein Isothiocyanate	FITC	Biolegend
CCR5	J418F1	monocytes	Fluorescein Isothiocyanate	FITC	Biolegend
